# Increased urinary sodium excretion is associated with systolic blood pressure in first degree relatives of hypertensive patients in Ibadan, Southwestern Nigeria

**DOI:** 10.11604/pamj.2018.31.168.16611

**Published:** 2018-11-09

**Authors:** Samuel Ajayi, Adewole Adebiyi, Solomon Kadiri

**Affiliations:** 1 Nephrology Unit, Department of Medicine, University College Hospital and College of Medicine, University of Ibadan, Ibadan, Nigeria; 2 Cardiology Unit, Department of Medicine, University College Hospital and College of Medicine, University of Ibadan, Ibadan, Nigeria

**Keywords:** Hypertension, first degree relatives, urinars sodium excretion

## Abstract

**Introduction:**

Understanding the risk factors of hypertension has led to a better understanding of the pathogenesis, prevention and reduction in morbidity of hypertension. It is well known that offsprings of hypertensive parents have an increased risk of developing hypertension. It is therefore necessary to explore the physiological differences between normotensive patients with and without a positive family history of hypertension with respect to their urinary excretion of sodium.

**Methods:**

This study was carried out at the University College Hospital, Ibadan Nigeria, to determine if normotensive patients with a positive family history of hypertension are different with respect to their urinary excretion of electrolytes and blood pressure. It examined the relationship between 24-hour urinary excretion of sodium, chloride and potassium, urea and creatinine and blood pressure in subjects with and without family history of hypertension. It was a case-control study of sixty-two subjects: normotensive patients’ first degree relatives of primary hypertensive patients and normotensive patients without positive family history.

**Results:**

The mean (SD) systolic blood pressures for subjects with and without family history of hypertension were significantly different: 120.0(22.25) and 105.0(17.50) respectively, (p=0.001). The mean arterial blood pressures were significantly different: 86.4(10.2) mmHg and 80.1(8.1) mmHg respectively (p=0.010). The mean (SD) 24-hour urinary excretion of sodium for normotensive patients with and without positive family history of hypertension were 180.5 (45.50) mEq/L, and 156.0(36.25) mEq/L respectively. Systolic blood pressure and 24-hour urinary excretion of sodium was also higher in normotensive subjects with a positive family history of hypertension.

**Conclusion:**

Systolic blood pressure and twenty-four hour urinary excretion of sodium were higher in normotensive subjects with a positive family history of hypertension than in those without a family history of hypertension.

## Introduction

Hypertension remains a major health problem in Africa, being the commonest cardiovascular cause of morbidity and mortality in adult patients [1-3]. Understanding the risk factors of hypertension, has led to a better understanding of the pathogenesis, and more importantly, prevention and reduction in morbidity of hypertension. Risk factors in hypertension are both environmental and genetic. Environmental risk factors include excessive alcohol consumption, cigarette smoking, psychosocial stress, socio-economic status, increased body adiposity, and excessive consumption of salt. Genetic risk factors are mainly due to heritability. It is well known that offsprings of hypertensive parents have an increased risk of developing hypertension [4-6]. In the Nigerian survey the odds ratio for hypertension in the offsprings where both parents were hypertensive was 1.5, as against the offsprings of normotensive parents [7]. Therefore, there is an increasing likelihood that first degree relatives of hypertensive patients may develop hypertension. With respect to salt, the increased susceptibility may be due to abnormal salt handling by the kidney and increased salt intake [8-10]. It is therefore necessary to explore the physiological differences between normotensive patients with and without a family history of hypertension with respect to their urinary excretion of sodium. This study examined the relationship between 24-hour urinary excretion of sodium, chloride and potassium, urea and creatinine and blood pressure in Nigerian subjects with and without family history of hypertension.

## Methods

This was a case-control study of sixty-two black, Nigerian subjects: 32 normotensive patients who were first degree relatives of primary hypertensive patients (Group I), 30 normotensive patients without positive family history of hypertension (Group II). Subjects with pregnancy, diabetes mellitus, evidence of any renal disease, any endocrine disorder, use of traditional medicine, and Body Mass Index (BMI) greater than 30 kg/m^2^ were excluded. The selection criteria for the normotensive groups followed the criteria specified by Watt [11]. All subjects recruited could give informed knowledge about blood pressure of all their first-degree relatives in the last six months, lived with their relatives and so could have them examined at home or brought to hospital. Patients with primary hypertension who were being followed up in the clinic were persuaded to bring first degree relatives to the clinic. Subjects who were 30-60 years old were included if they had normal blood pressure taken on two occasions within a week's interval. They were excluded if they were on medications or sick. In addition, death of any first degree relative from unknown causes was an exclusion criterion. Renal ultrasound scan, chest X-ray, urinalysis, blood sugar estimation and electrocardiogram were done in all hypertensive patients and their relatives at the screening stage, and though there were many with abnormal results, only those with "normal" results were enrolled into the study. Weight was measured with subjects in light clothing and without shoes on a beam type balance scale calibrated with standard weights. BMI was then calculated using the formula: *BMI = Weight (Kg)/Height(m^2^)*. Hypertension was diagnosed as blood pressure of ≥140/90mmHg. Urine collection: a clean, four-litre jar containing boric acid was given to all participants for urine collection for the period 7.00 a.m. of that day to 7.00 a.m. of the next day.

Each subject was asked to choose a convenient day when a 24-hour collection could be guaranteed. In most cases, Sunday morning 7.00 a.m. to Monday 7.00 a.m. was chosen so that subjects could still go work after bringing in their urine samples. At 7.00 a.m., the bladder was emptied whether the subject had the urge to void or not and the urine discarded. All urine passed subsequently till 7.00 a.m. next day was collected, and subjects were instructed to empty their bladder at 7.00 a.m. whether they felt the urge to void or not; this was the last urine collected. Only one 24-hour urine sample was collected as this has been shown to reflect the sodium and potassium intake over the previous 3 to 4 days [12]. On the following day when the collected urine was returned, they were questioned again to ascertain the method of urine collection. An aliquot of 20mls was then taken for analyses. Urine samples were discarded when unsatisfactory explanations were given. Also, samples measuring below 600ml were rejected [13] and the urine collection procedure repeated on another day. Subjects were asked not to change their dietary and drinking habits during the period of urine collection. The samples obtained were therefore likely to be an adequate qualitative and quantitative representation of their normal urine. It is not expected that there would be a wide variation in the nature of diets of participants. The total volume was measured and recorded and 10ml aliquot collected in heparinised tubes was analysed for Na+, K+, Cl-, HCO3-, urea and creatinine. Blood samples were also taken for serum electrolytes, urea and creatinine. Samples were analysed at the chemical pathology department of the hospital according to standard procedures. Ethics approval was obtained from the University College Hospital Research Ethics committee and all participants were duly consented at enrollment into the study. Patients who participated into the study were seen privately and the study was explained to them. They were free to refuse participation without any prejudice to their care in hospital. Their records were anonymized and were only identified by a study serial number. Electronic records and paper trails were secured and only accessible to the investigator or authorized trained personnel.

**Statistical analysis:** Data was entered into the Epi Info and then exported to R statistical package for analysis. The means, standard deviations (SD) were computed. The distribution of urinary variables was done by Shapiro-Wilk test for normality of data and t-test was thereafter used for analysis. Statistical significance was set at p< 0.05.

## Results

**Demographic and clinical data:** There were 29 (46.8%) males and 33 (53.2%) females. The mean (SD) age were 39.0(9.75) and 36.0(11.25) years in the two study groups respectively (Table 1). The mean (SD) systolic blood pressures for Groups I and II 120.0(22.25) and 105.0(17.50) respectively, and this was significantly different (p=0.001). The diastolic blood pressures were 70.3(9.1) and 66.7(7.4) mmHg respectively. The mean arterial blood pressures were 86.4(10.2) mmHg and 80.1(8.1) mmHg respectively. This was also significantly different (p=0.010). The mean (SD) BMIs were 23.1(5.55) and 22.3(3.70) kg/m^2^ for groups I and II respectively. The mean (SD) 24-hour urinary excretion of sodium for normotensive patients with positive family history of hypertension was 180.5 (45.50) mEq/L, and 156.0(36.25) mEq/L for normotensive patients without a family history. There was no significant difference in urinary potassium and chloride between the two groups. Results for the serum and urine parameters are shown in Table 2. The serum sodium, chloride, bicarbonate, urea, creatinine, creatinine clearance and urinary sodium-potassium ratio were not statistically different in the two groups. However, serum potassium and urinary sodium were higher in those with family history of hypertension and statistically different in the two groups 3.83(0.398)mEq vs 3.63(0.30)mEq/l and 180.5(45.50)mEq/l vs 156.0(36.25)mEq/l respectively.

**Table 1:** Demographic and clinical data of the study groups

Variable	Family history of hypertension (Group I)	No family history of hypertension (Group II)	P-value
**Sex:**			0.783
Male (%)	16(50.00)	13(43.30)	
Female (%)	16(50.00)	17(56.70)	
Age (years)	39.0(9.75)	36.0(11.25)	0.799
BMI (kg/m^2^)	23.1(5.55)	22.3(3.70)	0.098
Systolic BP (mmHg)	120.0(22.25)	105.0(17.50)	0.001
Diastolic BP (mmHg)	70.3(9.05)	66.7(7.43)	0.095
MAP (mmHg)	86.4(10.20)	80.1(8.10)	0.010

**Table 2:** Comparison of blood pressure and serum and urinary sodium and potassium in normotensive patients with or without family history of hypertension

Variable	Family history of hypertension (Group I)	No family history of hypertension (Group II)	P-value
Serum sodium (mEq/L)	138.0(3.00)	136.0(4.50)	0.074
Serum potassium (mEq/L)	3.83(0.40)	3.63(0.30)	0.030
Serum chloride (mEq/L)	103.4(3.25)	102.3(3.01)	0.169
Serum bicarbonate (mEq/L)	21.7(1.91)	21.8(1.96)	0.771
Serum urea (mEq/L)	21.5(11.00)	20.0(9.00)	0.386
Serum creatinine (µm/L)	1.00(0.30)	0.95(0.200)	0.808
Urinary sodium* (mEq/L)	180.5(45.50)	156.0(36.25)	0.034
Urinary chloride	137.9(44.18)	132.6(52.90)	0.669
Urinary potassium (mEq/L)	49.6(21.12)	45.3(15.35)	0.367
Urinary urea* (mEq/L)	4274.0(3782.75)	4489.5(2704.00)	0.213
Urinary creatinine (µm/L)	1079.1(366.50)	1190.3(429.21)	0.276
Creatinine Clearance (ml/min)	80.4(17.47)	87.0(20.92)	0.179
Urinary Na:K ratio	3.55(1.94)	3.60(1.77)	0.418

## Discussion

The higher mean systolic blood pressure (SBP) and mean arterial pressure (MAP) in offsprings of hypertensive patients is comparable and this is consistent with findings of other studies which have suggested that there is a gradual increase in blood pressure in those with family history of hypertension. This may also imply that there is an impairment of arterial compliance and increased sympathetic activity [14-16]. For instance, Zhou, *et al*, have demonstrated that normotensive offsprings of hypertensive parents had increased BP and impaired compliance of small and large arteries [17]. Indeed, family history is increasingly being recognised as a non-modifiable risk factor for metabolic syndrome, and this includes hypertension [18, 19]. The mean 24-hour urinary sodium excretion in the present study compares with previous studies [20, 21]. In this study, the 24-hour urinary sodium excretion was higher in normotensive with a positive family history of hypertension than in those without. This is in agreement with previous findings of increased sodium excretion and high blood pressure, especially systolic blood pressure [20-23]. The urinary excretion of potassium was not different in the two groups. A probable explanation is that those at risk of hypertension are believed to have higher salt taste thresholds than controls and so, are less able to discriminate a salty taste. This results in a higher consumption of salt than controls. This has been documented in both Caucasians and Africans [24]. Thus Obasohan, *et al*, concluded that the increased urinary output of salt may simply reflect intake rather than renal sodium handling abnormalities. Another explanation could be an inherent kidney abnormality even with a normal salt load. For increased salt intake to result in permanent changes in arterial pressure, renal function must be altered [25]. An increase in salt intake would lead to an increase in blood pressure and vice-versa, increased sodium reabsorption in the proximal tubule, and damage to the vascular wall as documented in previous studies [22, 26, 27].

Theories proposed to explain the blood pressure effect of salt loading on the blood pressure include an alteration of renal sodium handling. A sustained tendency to renal sodium and water retention leads to sustained long-term autoregulation eventually generating permanent elevation in peripheral vascular resistance with renal vascular lesions, and ultimately hypertension [28]. The overall consequence is a reduced renal plasma flow which leads to sodium retention. In the early stages of hypertension, there are varying degrees of reduced renal blood flow but with relatively well-preserved glomerular filtration rate. These abnormalities include reduced glomerular ultrafiltration coefficient, reduced GFR, reduced single nephron filtration and increased sodium reabsorption in the proximal tubule. The net result is that the amount of sodium delivered to the distal tubule is reduced [29]. Consistent with this hypothesis, diminished capacity for sodium and water excretion has been observed in models of hypertension. In those who develop hypertension, the blood pressure does not return to normal, possibly due to vascular damage and diastolic dysfunction [26]. Chronic but mild hypervolemia and hypertension could be the long-term consequences of reduced sodium excretion [29], and this may explain the comparatively higher systolic blood pressure in normotensive patients with positive family history of hypertension who are at a higher risk of developing high blood pressure. This defect of sodium excretion has also been used to explain the pathogenesis of the nephrotic syndrome [30, 31]. Contrary to earlier studies in which chloride was thought to play as much a role in hypertension as sodium [32-34], the 24-hour urinary chloride did not differ significantly in the present study groups. There was also no significant difference in 24-hour urinary potassium or in the ratio of urinary sodium/potassium. This study may provide and argument for measuring sodium excretion in the evaluation of individuals predisposed to hypertension and in population studies. Whilst a 24-hour measurement may be difficult and impossible for routine use, a spot-urine method has been validated for possible routine use [35]. We measured 24-hour urinary sodium which is regarded as the gold-standard. This is a strength of our study. The limitation was that this was done only once even though multiple measurements would have provided a more accurate reflection of daily intake of sodium.

## Conclusion

Systolic blood pressure and twenty-four hour urinary excretion of sodium was higher in normotensive subjects with a positive family history of hypertension than in those without a family history of hypertension. The possibility of using this finding as a screening tool needs to be explored further in this environment where scarce resources compel a need to emphasize prevention and anticipation of health problems rather than the curative aspects. Development of such a strategy affords the clinician an opportunity to intervene early (dietary and life style counselling) to delay or prevent the onset of high blood pressure in due course. Larger community-based studies are required to clarify more fully the relationship between sodium excretion, family history of hypertension and blood pressure.

### What is known about this topic

- Offsprings of hypertensive parents have an increased risk of developing hypertension;
- Increased susceptibility is due to intake and abnormal salt handling by the kidney.

### What this study adds

- The 24-hour urinary sodium excretion was higher in normotensive with a positive family history of hypertension than in those without;
- Systolic blood pressure and twenty-four hour urinary excretion of sodium was higher in normotensive subjects with a positive family;
- This study corroborates the theory of abnormal renal sodium handling in Black Nigerian individuals who are prone to developing hypertension perhaps through pressure natriuresis: that is, a higher blood pressure is required to excrete a given salt load.

## Competing interests

The authors declare no competing interests.
